# Manipulating PARK7/DJ-1 Levels by Genotoxic Stress Alters Noncoding RNAs and Cellular Homeostasis

**DOI:** 10.3390/cells14231860

**Published:** 2025-11-25

**Authors:** Keren Zohar, Haya Zoubi, Michal Goldberg, Tsiona Eliyahu, Michal Linial

**Affiliations:** 1 Department of Biological Chemistry, Institute of Life Sciences, The Hebrew University of Jerusalem, Jerusalem 9190401, Israel; keren.zohar@mail.huji.ac.il (K.Z.);; 2 Department of Genetics, Institute of Life Sciences, The Hebrew University of Jerusalem, Jerusalem 9190401, Israelgoldbergm@mail.huji.ac.il (M.G.)

**Keywords:** RNA-seq, regulated cell death, lncRNA, oxidation stress, X-ray, ribosome stability, miRNAs, siRNA, Parkinson’s disease

## Abstract

**Highlights:**

**What are the main findings?**

- DJ-1/PARK7 modulates the transcriptional response to X-ray-induced DNA damage, linking redox regulation to genome stability.
- Following X-ray irradiation, DJ-1 overexpression leads to coordinated suppression of the translation machinery and mitochondria, while DJ-1 knockdown disrupts global transcriptional homeostasis.

**What are the implications of the main findings?**

- DJ-1 acts as a key factor during genotoxic stress, integrating transcriptional coding and non-coding RNA networks.
- Understanding DJ-1-dependent pathways may support the development of targeted strategies to enhance maintaining genomic integrity.

**Abstract:**

DJ-1/PARK7 is a multifunctional protein that plays a vital role in sensing oxidative stress and maintaining redox homeostasis. As an oncogene, DJ-1 influences p53-mediated stress responses and contributes to cancer progression. This study investigates the impact of X-ray-induced DNA breaks on cellular responses under varying DJ-1 expression levels. Using siRNA knockdown and overexpression approaches, transcriptional changes were analyzed by RNA-seq. Naïve cells exhibited only a moderate response to X-ray exposure, including suppression of the cell cycle and activation of stress pathways. In contrast, DJ-1 overexpression caused pronounced gene-expression suppression, particularly affecting ribosomal genes and mitochondria, with 21- and 3.5-fold enrichment, respectively. DJ-1 knockdown led to extensive, non-specific transcriptional changes affecting ~18% of all transcripts (~3400), indicating disrupted cellular homeostasis. Under DJ-1 knockdown, X-ray irradiation resulted in a 3.7-fold enrichment of suppressed DNA-damage response genes. Notably, approximately 25% of non-coding RNAs (ncRNAs) were differentially expressed following DJ-1 manipulation. X-ray-irradiated cells with DJ-1 overexpression also showed reduced expression of SNHG lncRNAs that host snoRNAs, potentially altering miRNA-sponging capacity and ribosomal regulation. These findings underscore DJ-1’s critical role in modulating cellular responses to genotoxic stress, reshaping transcriptional landscapes, and regulating ncRNA profiles. The dual impact of DJ-1 on redox and transcriptional networks positions it as a potential therapeutic target in diseases involving oxidative stress and impaired DNA repair.

## 1. Introduction

PARK7 (DJ-1) is a multifunctional protein implicated in a variety of human diseases [1,2,3]. DJ-1 is expressed in cells and tissues to scavenge reactive oxygen species (ROS). It also acts as a chaperone, regulates transcription, and is involved in signal transduction and mitophagy [4]. DJ-1 was discovered as a causative gene for Parkinson’s disease (PD) [5] and other age-related diseases [6]. Beyond its direct role in protecting neurons from oxidative damage, DJ-1 has also been implicated in inflammatory [3] and metabolic diseases [5]. Depletion of DJ-1 in pancreatic beta cells reduces their capacity to cope with stress and makes them prone to diabetes [7]. Manipulations of DJ-1 in animal models confirmed its causal role in obesity-induced inflammation [8], metabolic reprogramming and energy homeostasis [9,10].

At the molecular and cellular levels, the human PARK7 gene encodes DJ-1, a ~20 kDa protein that functions as a homodimer. The dimer is predominantly localized to mitochondria, where it contributes to the regulation of electron transport, apoptosis, and mitophagy [11]. As a sensor of oxidative stress, DJ-1 protects cells from ROS-induced damage [4,12], and its activity is regulated by the oxidation status of key cysteine residues [13]. Upon oxidative stress, DJ-1 scavenges ROS and participates in mitochondrial quality-control pathways [14]. Its neuroprotective effect is attributed to its major antioxidant function [15], particularly through the Keap1–Nrf2 pathway, which governs cellular redox homeostasis [16]. Under oxidative stress, DJ-1 activates and stabilizes Nrf2 by dissociating it from Keap1 and preventing its ubiquitination. By promoting nuclear translocation of Nrf2, DJ-1 induces the expression of numerous antioxidant and detoxifying genes. When DJ-1 is depleted, antioxidant enzyme levels decline and apoptosis or other regulated cell-death pathways are triggered [17].

In addition to its well-established role in oxidative stress responses, DJ-1 is also recognized as a mitogen-dependent oncogene that promotes the progression of various cancers. High DJ-1 expression is linked to poor prognosis across numerous cancer types, including brain tumors, breast cancer, non-small cell lung carcinoma, prostate cancer, pancreatic carcinoma, hepatocellular carcinoma, and colorectal cancer. In most cases, elevated expression correlates with aggressive disease, reduced survival, and metastasis [18]. Several mechanisms through which DJ-1 supports tumor progression have been proposed. For example, stable interaction of DJ-1 with anti-apoptotic factors (e.g., Bcl-Xl) can prevent the release of mitochondrial factors such as cytochrome c and apoptosis-inducing factor (AIF) into the cytoplasm [19]. A functional link between p53 and DJ-1 has been proposed in the context of oxidative stress and through DJ-1’s nuclear activities. Specifically, DJ-1 may mediate the activation of p53 in response to oxidative stress, while loss of p53 has been suggested to increase DJ-1 expression in transformed cells, indicated by oncogenic activation of AKT. DJ-1 overexpression is also associated with inhibition of tumor suppressors such as PTEN, thereby activating the PI3K/AKT signaling pathway and promoting cell growth and survival, which underlies the poor prognosis in many cancers [20].

The response of DJ-1 to oxidative stress and elevated ROS is well established. In addition to the physiological production of ROS by active mitochondria, X-ray irradiation generates hydroxyl radicals and hydrogen peroxide, which at high levels cause damage to DNA, lipids, and proteins. Whether DJ-1 participates in the DNA damage response (DDR) remains unknown. Following X-ray irradiation, cells activate the DDR, a network that senses double-strand breaks (DSBs) and initiates repair mechanisms [21]. DDR activation primarily arrests DNA replication to allow repair by mechanisms such as homologous recombination (HR) and non-homologous end joining (NHEJ). When damage is excessive, cells eventually undergo programmed cell death, senescence, or other regulated cell-death pathways [22]. A recent study suggested that pathological mutations in DJ-1 impair DNA repair activity in PD patients [23]. The proposed model is that unrepaired DNA damage contributes to PD pathology; throughout disease progression, neurons with defective DNA repair accumulate genome instability, mitochondrial dysfunction, and ultimately neurodegeneration.

In this study, we asked whether we could detect a distinct cellular response and a shift in cell-fate decisions following X-ray irradiation under controlled conditions in which DJ-1 expression levels were experimentally manipulated. Specifically, we examined the effects of reducing DJ-1 (KD), increasing DJ-1 (OX), or maintaining baseline expression, and monitored the corresponding cellular responses. We identified global transcriptional changes as well as consistent alterations in the abundance and diversity of ncRNAs, particularly lncRNAs. Changes in cellular homeostasis, regulated cell death, and translation were observed in cells challenged with X-ray irradiation under varying DJ-1 baseline levels.

## 2. Materials and Methods

### 2.1. Cell Viability Using Flow Cytometry (FACS)

Human embryonic kidney 293 cells (HEK293, ATCC) were cultured in 6-well plates using Dulbecco’s modified Eagle’s medium high-glucose supplemented with 10% fetal bovine serum. Cells were grown to 70–80% confluence at 37 °C and 5% CO_2_ before analysis by fluorescence-activated single-cell sorting (FACS). HEK293 cells were incubated with the fluorescent dye propidium iodide (PI) and Annexin V-FITC, purchased from MBL (MEBCYTO-Apoptosis Kit; Woburn, MA, USA). Cells were stained with Annexin V according to the manufacturer’s protocol. Annexin V fluorescence indicates the presence of phosphatidylserine on the outer leaflet of the plasma membrane. Apoptosis and other phases of cell death were discriminated by staining with PI and Annexin V [24,25]. After staining, live cells show little or no fluorescence, while apoptotic cells exhibit FITC fluorescence of Annexin V) [26]. FACS analysis was conducted with 30,000 cells, and the fraction of PI-positive dead cells was determined.

### 2.2. Knockdown by siRNAs

Cells were cultured in a 6-well plate at 20–30% confluence (3 × 10^4^ per cm^2^). For transfection, Lipofectamine 2000 (Lipo2000; Invitrogen, Cat #11668019, Carlsbad, CA, USA) was used in cells at 40–60% confluence, following the manufacturer’s instructions. Lipo2000 is considered optimal for HEK293 cells [27,28]. The esiRNA (MISSION system, Sigma-Aldrich, Burlington, MA, USA) consists of a pool of hundreds of 21 bp siRNAs, where each individual dsRNA is present at low concentration to minimize off-target effects while maintaining efficient knockdown (KD). We applied esiRNA-RULC, a mixture of 21 nt dsRNAs used as a negative control (RLUC refers to esiRNA directed against Renilla luciferase; EHURLUC), alongside the specific PARK7 esiRNA (to knockdown DJ-1 transcripts and protein; EHU113961). RULC experiments were used to measure baseline effects of esiRNA delivery and to distinguish cellular responses to the targeted siRNA itself [29]. After 18 h, cells were exposed to X-ray radiation (parameters: 12.5 mA, 320 kV, 10 Gy). Six hours later, RNA was extracted.

### 2.3. Overexpression of DJ-1

One day prior to transfection, cells were plated in a 10 cm plate at an initial density of 5 × 10^6^ cells per plate. The pCMV3 vector encoding canonical DJ-1, fused to double Strep-tags at its C-terminus, was obtained from Sino Biological. The Strep-tag peptide exhibits a high intrinsic affinity for Strep-Tactin, an engineered streptavidin. The expression plasmid (DJ-1 or empty pCMV3 vector) and PEI (260008-5; Polysciences, Warrington, PA, USA) were separately diluted in Opti-MEM I (31985-047; Gibco, Grand Island, NY, USA), then mixed and incubated at room temperature (RT) for 25 min to allow polyplex formation prior to addition to the cells. The mixture was added dropwise onto the cell culture. After 18 h, cells were exposed to X-ray radiation (parameters: 12.5 mA, 320 kV, 10 Gy). RNA was extracted six hours later.

### 2.4. Western Blot

Cells were lysed using 2× SDS with DTT lysis buffer, incubated at 95 °C for 5 min, and stored at −20 °C. Before use, lysates were re-heated at 95 °C for 5 min, followed by a brief spin to remove debris. Protein lysates were electrophoresed on 13% SDS-polyacrylamide gels and then transferred semi-dry to nitrocellulose membranes. Membranes were blocked with TBS-T buffer (0.15 M NaCl, 0.05 M Tris, 0.1% Tween-20, pH 7.6) containing 5% skim milk (BD Difco) at RT for 30 min. Membranes were washed with TBS-T and incubated overnight at 4 °C with primary antibodies diluted according to the manufacturer’s instructions in 4% BSA. After washing with TBS-T, membranes were incubated with secondary antibodies (1:1000 in TBS-T with 4% skim milk) for 1 h at RT, followed by three washes with TBS-T, 10 min each. Chemiluminescence was visualized using an ECL detection kit (Biogate, Ness Ziona, Israel) with a gel imager (ChemiDoc, Bio-Rad, Hercules, CA, USA, or Fusion FX, Viber Lumart, Marne-la-Vallée, France) and quantified using Image Lab 6.1. Results were normalized to the loading control (β-tubulin).

### 2.5. Reverse Transcription PCR (RT-PCR) and PCR

Cells were collected for RNA preparation. Total RNA was extracted from cell cultures using TRIzol (Thermo Fisher Scientific, Waltham, MA, USA), and reverse transcription was performed using a Ready-To-Go first-strand synthesis kit (Cytiva, Marlborough, MA, USA) according to the manufacturer’s instructions. One microgram of RNA was reverse transcribed into cDNA and used in PCR reactions. The PCR conditions consisted of an initial denaturation at 95 °C for 2 min, followed by 35 cycles of 10 s at 95 °C, 15 s at 60 °C, and 30 s at 72 °C, with a final extension of 5 min. PCR products were separated on a 1.5% agarose gel, stained with ethidium bromide, and quantified by densitometry using ImageJ (Ver 1.54, GitHub). The primers were designed against human RefSeq by the Primer3Web tool (Ver 4.1.0). The forward (F) and reverse (R) primers of β-actin (196 nt), F:CATGCCCACCATCAGCCCTGG and R:ACAGAGCCTCGCCTTTGCCGA. For DJ-1 (376 nt): F:GCCTGGTGTGGGGCTTGTAA and R: GCCAACAGAGCAGTAGGACC. For DJ-1, which is only suitable for transcripts from the plasmid (and not endogenous DJ-1) (447 nt): F:CAGTGTAGCCGTGATGTGGT and R:AGCAGACCCCGCGTCTTTA. Several ncRNAs were tested: SNHG15 (NR_152597.1), F:CCTCTCACCAGTGGCTTCAC and R: GGCAACAAGCGAGGTTTCAA (276 nt); MINCR (NR_120682.1), F: TCAGGCTTCCGGTCTGTTTG and R: TGCCACATGGCACAGTATCT (322 nt) and GAS5/SNHG2 (NR_186288.1), F: CCTGTGAGGTATGGTGCTGG and R: AGCTGCATGCTTGCTTGTTG (225 nt).

### 2.6. RNA-Seq

Total RNA was extracted using the RNeasy Plus Universal Mini Kit (QIAGEN, Cat #73404, Redwood City, CA, USA) according to the manufacturer’s protocol. One microgram of total RNA was used for poly (A) selection to enrich for mRNAs. Libraries were prepared with the KAPA Stranded mRNA-Seq Kit following the manufacturer’s instructions and sequenced on an Illumina NextSeq 500 platform to generate 85 bp single-end reads, yielding 25–30 million mapped reads per sample.

### 2.7. Bioinformatic Analysis and Statistical Analysis

Next-generation sequencing (NGS) data underwent quality control using FastQC (v0.11.9), followed by preprocessing with Trimmomatic (v0.32). Reads were aligned to the human reference genome GRCh38 (hg38) using STAR (v2.7.0a) with default parameters. Data were normalized to TPM (transcripts per million) to account for gene length and enable comparison between genes. Summary statistics for the quality control listed in Supplementary Table S1. Genomic loci were annotated with GENCODE release 46. Lowly expressed genes were filtered out using a threshold of at least two counts per million in three samples.

Differential expression (DE) analysis was performed for all experimental groups. Genes with FDR < 0.05 and an absolute log fold change (FC) ≥ |0.5| were considered up- or downregulated, respectively; all other genes were considered unchanged. DE genes (DEGs) were partitioned into coding (cod) and non-coding (nc) transcript biotypes (including pseudogenes, antisense RNAs, miRNAs, TEC, lncRNAs, and other rare categories). Because PARK7 overexpression accounted for ~4.5% of all normalized reads, we removed PARK7 expression and recalibrated the data prior to differential expression analysis. Gene expression normalization (nTPM) was used to define basal DJ-1 levels in HEK293. Principal component analysis (PCA) was performed using the R base function prcomp (RStudio v4.1.0). Differential expression analysis was conducted with edgeR (v3.36.0), using trimmed mean of M-values (TMM) normalization. Multiple comparisons were corrected using the false discovery rate (FDR) threshold of 0.05. edgeR was selected for its robustness to deviations from normality. Figures were generated using the ggplot2 R package (v3.3.5).

Functional and network analyses were performed using STRING, with a minimum protein–protein interaction (PPI) confidence score of 0.7. For ncRNA functional enrichment, we used LncSEA 2.0, which incorporates > 400 k reference lncRNA sets across 33 annotated functional categories [30]. These annotations integrate TF ChIP-seq, DNase-seq, ATAC-seq, and H3K27ac ChIP-seq datasets from hundreds of human cell types. The Simpson index was used to assess the functional diversity of lncRNAs (lower values indicating concentration in fewer functions). RNA–protein interactions were assessed using integrated data from RNAInter 4.0, NPInter 4.0, and ENCORI. Indirect connectivity among lncRNAs, genes, and miRNAs was mapped using lncTAR 2.0 and ncPATH, incorporating ~32 k validated lncRNA–target interactions, ~5 k ceRNA networks, and ~0.5 M predicted coding-gene links across 220 KEGG pathways [31].

## 3. Results

### 3.1. DNA Damage Response by X-Ray Irradiation

**Figure 1:**
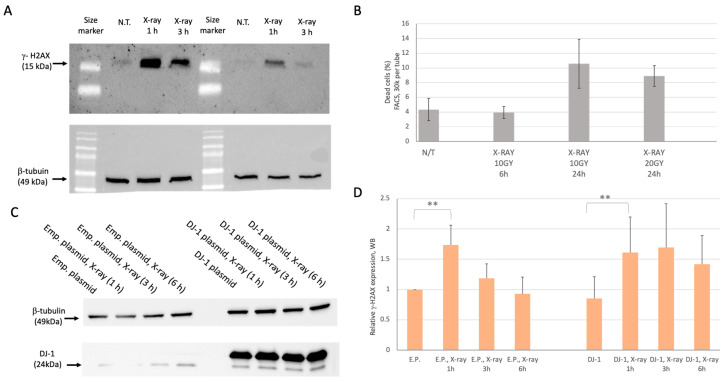
Establishing cellular conditions for X-ray and DJ-1 overexpression. (**A**) Western blot (WB) analyses using antibody to γ-H2AX with extract from naïve (non-treated; N.T.) cells, and following X-ray treatments, 1 h and 3 h post irradiations. Protein extracts were separated on 13% SDS-PAGE gel. The sensitivity of the WB was increased by partition the cell extracts to 80% (left) and 20% (right), with β-tubulin (49 kDa) served as internal controls for sample loading. (**B**) Average of the PI-based analysis in triplicates. There was no significant difference between the N.T. and X-ray 10 GY at 6 h (h). Viability of cells 24 h after irradiation was reduced. Note that cell viability was insensitive to the intensity of X-ray used for 10 or 20 Gy. (**C**) WB of the cells transfected with mock experiment (empty plasmid, left) at three time points from X-ray irradiation and cells transfected with OX of PARK7/DJ-1 plasmid. Note the lower band (24 kDa) represents endogenous DJ-1 and the OX with a higher Mw (27 kDa) due to the addition of C-terminal recombinant Strep tag. (**D**) Following the kinetics of the experiments as in C, the relative expression of the γ-H2AX was measured (in triplicates) indicating the statistically increased level 1 h post irradiation (10 Gy) in cells with empty plasmid (left) and DJ-1 OX (right). ** indicates *p* < 0.05 (two-tailed *t*-test).

To assess the impact of X-ray irradiation on cell-fate decisions under varying DJ-1 levels, we optimized experimental conditions to achieve an efficient DNA damage response (DDR) while minimizing cell death. Phosphorylation of histone H2AX on Ser139 (γ-H2AX) serves as a transient marker of local DDR at sites of double-strand breaks (DSBs) [32]. In a mock experiment with empty-plasmid overexpression, the maximal γ-H2AX signal was observed 1 h post-irradiation, followed by a decline in modified H2AX levels (Figure 1A). Radiation doses were calibrated, and cell survival was monitored by FACS using PI staining to quantify dead cells (see Section 2). In naïve, untreated cells (N.T.), the fraction of dead cells was minimal (~4%), with only a modest decrease in viability 24 h post-irradiation (Figure 1B). At this time point, cell survival remained approximately 88–90% (Figure 1B), and no increase in apoptosis was observed (Supplementary Figure S1). These results indicate that, although the DDR is robustly activated immediately after DNA damage (as indicated by γ-H2AX levels), long-term effects on apoptosis and cell death are minimal within 6 h post-irradiation

Figure 1C shows DJ-1 protein levels by WB. Endogenous DJ-1 was compared with and without X-ray treatment at 1, 3, and 6 h post-irradiation. Following overexpression (OX) of the PARK7/DJ-1 plasmid, a strong 27 kDa band was detected, representing a longer variant of DJ-1 due to the C-terminal Strep tag (Figure 1C, see Section 2). The strong signal from OX DJ-1 did not affect endogenous protein levels, which remained stable across all conditions, including 6 h after 10 Gy X-ray exposure. At each time point, the relative expression of γ-H2AX was measured. In cells transfected with empty plasmid (E.P.; Figure 1D, left) and in OX DJ-1 cells (Figure 1D, right), γ-H2AX increased within 1 h post-irradiation. In OX DJ-1 cells, the decay at 6 h was not significant, suggesting that overexpression may subtly alter DDR kinetics. Overall, γ-H2AX, a fast-responding marker of DSBs, reached maximal levels 1 h after genotoxic stress, while cell viability remained unchanged at 6 h, allowing assessment of the accumulated outcomes of irradiation.

### 3.2. Testing the Impact of X-Ray Irradiation in Cells Ranges by Different Levels of DJ-1

**Figure 2:**
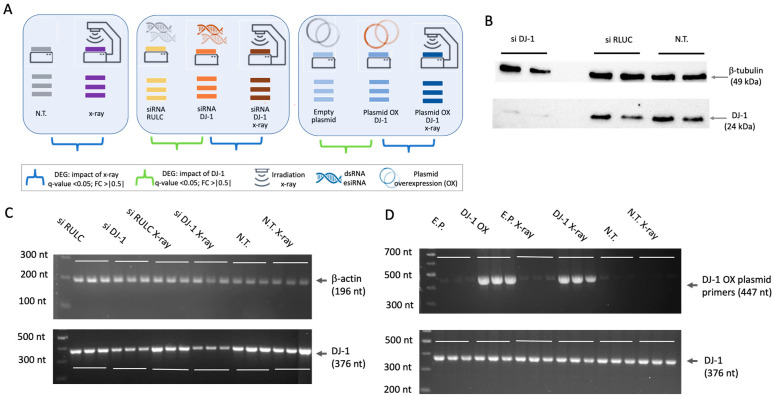
Comparative RNA-seq experiment in HEK293 cells with different baseline levels of DJ-1 following X-ray exposure (10 Gy, 6 h post-irradiation). (**A**) Schematic of the experimental setup for RNA-seq. Each group includes biological and technical triplicates, with or without X-ray treatment (10 Gy). Pairs of experimental groups are indicated by blue and green parentheses. The blue pairs are discussed in the main text (6 conditions, 18 RNA-seq libraries). Differentially expressed genes (DEGs) were defined as those with log2|FC| > 0.5 and adjusted *p*-value (FDR) < 0.05. (**B**) WB of DJ-1 KD cells. Three conditions were analyzed: DJ-1 siRNA, non-specific siRNA (RULC), and naïve (N.T.) controls. Protein extracts were separated on a 10% SDS-PAGE gel. β-tubulin served as a loading control, and DJ-1 antibody detected endogenous DJ-1 levels. (**C**) RT-PCR of treated cells for the indicated conditions. PCR products were separated on an agarose gel. DJ-1 amplicons are shown with β-actin as an internal control. Results for untreated (N.T.) and DJ-1 KD cells are presented. (**D**) RT-PCR for DJ-1 OX cells (transfected with DJ-1 plasmid) and N.T. Top: primers specific for OX DJ-1 plasmid. Bottom: primers specific for endogenous DJ-1 (see Section 2).

Exposing living cells to X-ray induces double-strand breaks (DSBs) and, as a byproduct, increases oxidative stress. To study the transcriptomic response to X-ray irradiation, we manipulated baseline DJ-1 levels using knockdown (KD) and overexpression (OX) protocols. Figure 2A illustrates the experimental design, in which cells were irradiated while DJ-1 expression was altered. We examined HEK293 cells under naïve conditions (N.T.), with DJ-1 knockdown (DJ-1 KD), and with DJ-1 overexpression (DJ-1 OX). All experiments were conducted under identical conditions to assess short-term responses 6 h post-10 Gy irradiation, with normalization applied as described in Section 2. We focused on transcripts, both coding and non-coding, that were significantly altered by X-ray treatment.

Figure 2B shows a WB of the KD experiment. DJ-1 protein levels were negligible after siRNA treatment, while non-specific siRNA (siRNA RULC, see Section 2) had no effect on basal DJ-1. Protein suppression was estimated at 5–10-fold. Figure 2C shows RT-PCR results from cells collected 6 h post-irradiation. Consistent with protein data, DJ-1 siRNA effectively reduced DJ-1 transcripts, whereas siRNA RULC had no effect. Similarly, naïve cells (N.T.) and X-ray-treated cells showed stable endogenous DJ-1 transcript levels. These results confirm that the KD protocol is specific and efficiently reduces DJ-1 expression.

Figure 2D shows RT-PCR results for the OX experiment. Primers specific to the introduced DJ-1 plasmid detected a strong signal in OX cells compared with empty-plasmid controls. Notably, endogenous DJ-1 levels remained unchanged, consistent with the protein data, demonstrating that overexpression specifically increased exogenous DJ-1 without altering the native protein.

### 3.3. Global Statistics of Transcriptomes of Cells Exposed to X-Ray

**Figure 3:**
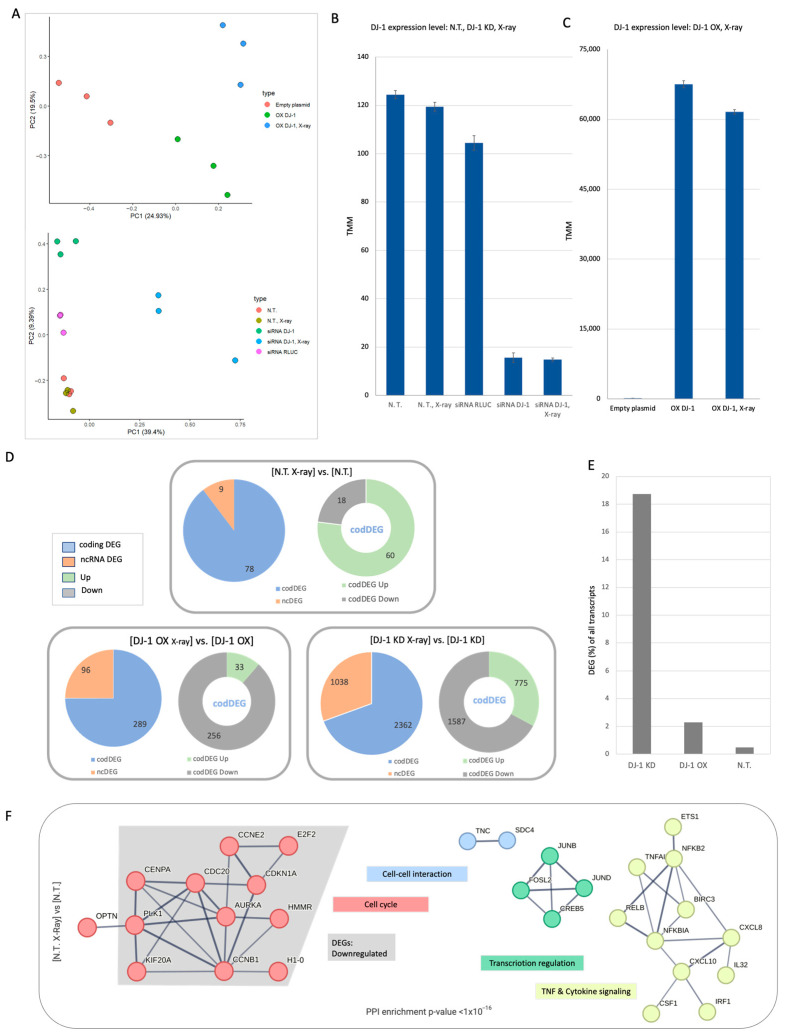
Experimental clustering and transcriptome view of cell response to X-ray irradiation. (**A**) PCA of cell samples based on differentially expressed genes (DEGs). Top: each cellular setting is colored by experimental group (empty plasmid, DJ-1 OX, and DJ-1 OX + X-ray). PC1 and PC2 together explain 44% of the variance. Bottom: five cellular settings colored by experimental group. PC1 and PC2 account for 48.8% of total variance. Note that N.T. and N.T. + X-ray overlap in the PCA, indicating minimal separation. Each sample is represented by biological triplicates. (**B**) Expression of DJ-1 transcripts across N.T., DJ-1 KD with and without X-ray, and siRNA RULC as a non-specific control. Expression levels are shown after TMM normalization. (**C**) Expression of DJ-1 transcripts across DJ-1 OX cells with and without X-ray. An empty-plasmid control is included. (**D**) Summary of DEG statistics before and after X-ray irradiation for N.T., DJ-1 OX, and DJ-1 KD. Significant DEGs are labeled as up- or downregulated (log2|FC| > 0.5) for each condition relative to matched controls. DEGs are divided into coding (codDEG, blue) and non-coding (ncDEG, orange), with upregulated genes in green and downregulated genes in gray. N.T., non-treated cells. (**E**) Fraction (%) of DEGs among all identified transcripts for N.T. and cells with manipulated DJ-1 levels (DJ-1 OX and DJ-1 KD). (**F**) STRING PPI network for codDEGs of N.T. X-ray vs. N.T. Only the subnetworks with ≥2 interacting genes are shown (*p*-value: <1.1 × 10^−16^; STRING confidence score ≥ 0.7). Functions associated with each subnetwork is color-coded. The gray background in the Cell cycle network highlights downregulated genes.

To assess the quality of the RNA-seq data, we analyzed normalized reads using principal component analysis (PCA, Figure 3A). Approximately 44% and 49% of the variance was explained by PC1 and PC2 for the DJ-1 OX and DJ-1 KD experiments, respectively. Cellular transcript levels of DJ-1 were quantified following its manipulation, with and without X-ray treatment. Figure 3B shows the RNA-seq results, including normalized endogenous DJ-1 levels and levels after KD. DJ-1 KD reduced the original transcript level by 8-fold [29], with no effect on basal DJ-1 levels in cells treated with non-specific siRNA RULC (see Section 2). Figure 3C shows results from all three conditions (each in triplicate) for the DJ-1 OX experiment. Overexpression of DJ-1, but not the empty-plasmid control, led to a dramatic increase in DJ-1 transcripts by at least 200-fold. These results indicate that the experiment is robust, with reliable clustering of biological triplicates across experimental groups.

Comparing the extent of differentially expressed genes (DEGs) across N.T., DJ-1 OX, and DJ-1 KD experiments revealed that the strongest overall response occurred in DJ-1 KD, affecting approximately 3400 genes, of which 30% were non-coding DEGs (ncDEGs) (Figure 3D). In contrast, in the N.T. setting, only 10% of DEGs were ncDEGs after X-ray irradiation, while the fraction of ncDEGs was 25% in the DJ-1 OX setting. The increases in ncDEG fractions in DJ-1 OX and DJ-1 KD were statistically significant (hypergeometric distribution *p*-values of 0.003 and 7.7 × 10^−29^, respectively). Focusing on ncDEGs, we observed roughly 10 times more genes in DJ-1 KD than in DJ-1 OX (1038 vs. 96) and 100-fold more than in N.T. cells (9 ncDEGs, Figure 3D). These results indicate that ncDEG representation varies substantially depending on cellular DJ-1 levels. Additionally, following X-ray exposure, the majority of coding DEGs (codDEGs) were downregulated (gray). In the DJ-1 OX condition, upregulated codDEGs (green) accounted for only 11% (33 genes). Supplementary Table S2 lists all codDEGs and ncDEGs along with their statistically significant and normalized fold change.

To assess the impact of X-ray on the transcriptional signature, we calculated the fraction of DEGs relative to background levels. Figure 3E shows that only 0.5% of genes were classified as DEGs in N.T. cells, whereas DJ-1 depleted cells (KD) exhibited a markedly stronger response, with >18% of genes identified as DEGs.

### 3.4. Naïve Cells Display a Limited but Coordinated Response to X-Ray Irradiation

Figure 3F shows the protein–protein interaction (PPI) network of the 78 coding DEGs (codDEGs) from the N.T. setting (STRING *p*-value < 1.1×10^−16^; confidence score ≥ 0.7). Among these, 60 genes were upregulated and 18 were downregulated (Figure 3D). Notably, the cell cycle-enriched subnetwork is almost entirely composed of downregulated DEGs (gray background, Figure 3F). Other subnetworks, including TNF signaling, transcription, and cell–cell interaction, are primarily composed of upregulated DEGs. The TNF signaling network is centered on RELB, NFKBIA, and NFKB2, which interact to form distinct transcriptionally active complexes that, together with NF-κB, orchestrate cell survival pathways (Figure 3F). NF-κB is a pleiotropic transcription factor involved in inflammation, differentiation, cell growth, tumorigenesis, and apoptosis. Consistent with these patterns, the cell cycle was suppressed, while TNF signaling, transcription, and cell–cell interaction pathways were actively induced in response to X-ray-induced genotoxic stress.

### 3.5. Downregulation in Ribosomal and Mitochondrial Transcripts Dominates DJ-1 Overexpression

**Figure 4:**
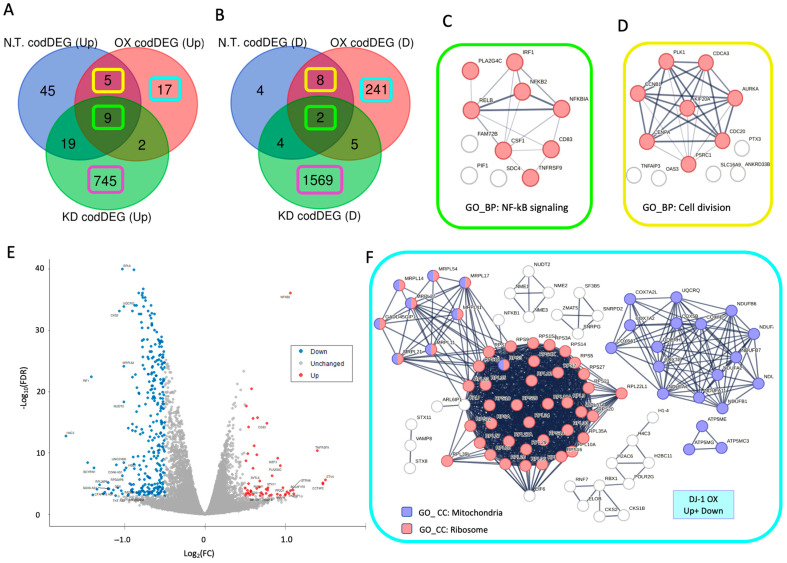
Analyzing the codDEG for X-ray irradiation, relative to non-irradiated cells. (**A**) Venn diagram of the upregulated codDEG (marked Up) for N.T., DJ-1 OX, and DJ-1 KD cell conditions. (**B**) Venn diagram of the downregulated codDEG (marked Down) for N.T., DJ-1 OX, and DJ-1 KD cell conditions. The shared genes between all three cellular settings are indicated by light green frame. Additional codDEG that overlap the N.T. and DJ-1 OX are colored by a yellow frame. The unique DJ-1 OX set is colored by light blue frame. (**C**) STRING-based PPI networks (confidence score ≥0.7) for the 11 shared DEGs by the green colored frames shown in Figure 3A,B. (**D**) STRING-based PPI networks (confidence score ≥0.7) for 13 DEGs, marked by the yellow-colored frames, shown in Figure 3A,B. (**E**) Volcano plot of the DJ-1 OX X-ray vs. cells with DJ-1 OX. The plot shows the fold change (FC) by log_2_(FC) and minus log10(FDR). (**F**) STRING-based PPI networks (confidence score ≥0.9) for the 258 unique DJ-1 OX DEGs by the light blue colored frames shown in Figure 3A,B. Only networks of ≥3 genes are shown. The colored nodes are annotated by the colored frame for the matched gene ontology cellular component (GO_CC) annotation. The connectivity of the STRING results for the shared genes is statistically significant (*p*-value < 1.0 × 10^−16^). The codDEG are listed in Supplementary Table S2.

The transcriptomes of cells with baseline (N.T.), overexpressed (OX), and depleted (KD) DJ-1 levels were compared based on trends of upregulated (Up, Figure 4A) and downregulated (Down, Figure 4B) genes. We observed that the overlap of codDEGs across the different cellular settings is limited. While the union of all codDEGs exceeds 2800, the fraction of genes shared among conditions is negligible (~2%), indicating that each setting exhibits a largely distinct transcriptional program (Supplementary Table S2). A striking observation was the exceptionally high number of codDEGs in DJ-1 KD (2362 genes). Despite this large number, very few overlap with the other two conditions, a finding that is statistically significant (hypergeometric test *p*-value: 6 × 10^−5^). Only 11 genes are shared among N.T., DJ-1 OX, and DJ-1 KD (Figure 4C, green frame), all belonging to the TNF and NF-κB signaling network. Additionally, 13 DEGs overlap between N.T. and DJ-1 OX cells (Figure 4D, yellow frame), forming a functional network enriched for cell division. These results indicate that, across all cellular settings following X-ray irradiation, cell division is suppressed while TNF and NF-κB signaling pathways are activated.

The transcriptomes from cells with a baseline level of DJ-1 (N.T.), overexpressed and depleted levels of DJ-1 were compared according to the trend of upregulated (Up, Figure 4A) and downregulated (Down, Figure 4B). We observed that the number of overlapping codDEG across the different cellular settings is limited. While the union of all codDEG in >2800, the number of genes at any intersection is negligible (2%), arguing for a unique transcriptional program for each setting (Supplementary Table S2). An unexpected observation concerns the exceptionally high number of codDEG in DJ-1 KD (2362 codDEG). Despite the very high number of DEGs, a negligible number of them overlap with the other two experimental settings. This observation is statistically significant (hypergeometric distribution test *p*-value: 6e-05). Still, 11 genes are shared between N.T., DJ-1 OX and DJ-1 KD (Figure 4C, green frame) belong to the network of TNF and NF-kB signaling. Additionally, the 13 DEGs overlap between the N.T. and DJ-1 OX cells (Figure 4D, yellow frame) forms a functional cell division enriched network. We concluded that in all cellular settings following X-ray irradiation, cell division, TNF and NF-kB signaling pathways are modulated.

Figure 4E shows a Volcano plot of DJ-1 OX cells after X-ray treatment versus untreated DJ-1 OX cells. The majority of genes were strongly downregulated (blue dots). However, a subset of significantly upregulated DEGs was associated with NF-κB signaling (as shown in Figure 4C). We next examined the nature of the unique codDEGs in the DJ-1 OX setting, which comprised 17 upregulated and 241 downregulated genes (Figure 4A,B). Analysis of these codDEGs (Figure 4F, light blue frame) revealed that a large fraction corresponded to ribosomal protein genes (Figure 4F, red nodes). The network further extended to mitochondrial ribosome genes (Figure 4F, nodes colored purple/red). Additionally, a tight smaller network of downregulated DEGs included genes involved in mitochondrial energy production (Figure 4F, purple nodes). Other downregulated subsets included a network of chromatin-modifying genes (10 genes, represented by H2AC6 and RBX1), as well as smaller subnetworks involving DEGs related to vesicle trafficking and secretion (e.g., VAMP8), splicing machinery (e.g., SF3B5), and members of the NME family (e.g., NME1–3). NME genes are implicated in diverse cellular processes, including energy metabolism, cytoskeletal dynamics, and DNA repair. For instance, NME1 is recruited to sites of DNA damage, contributes to proofreading, and influences the mode of DSB repair [33]. Collectively, these results indicate that 6 h after X-ray exposure, DJ-1 overexpression triggers a coordinated transcriptional response that suppresses major cellular processes, including chromatin-transcription regulation, energy production, and prominently the translation machinery.

Analysis of codDEGs from DJ-1 KD cells after X-ray treatment relative to DJ-1 KD alone identified a large number of significant genes (>2300). Despite this, the PPI network generated using STRING was relatively sparse (Supplementary Figure S2), and only broad functional enrichments were detected. Functional enrichment of the upregulated genes (775 genes, Figure 3D) highlighted cell junction and membrane functions, whereas downregulated codDEGs were primarily associated with nuclear processes and transcriptional regulation (Supplementary Figure S2). These findings may indicate that DJ-1-depleted cells undergo substantial transcriptional changes upon X-ray irradiation, potentially affecting nuclear function and chromatin-related processes. A detailed functional enrichment analysis of codDEGs for DJ-1 KD X-ray relative to DJ-1 KD is provided in Supplementary Table S3, along with the expression trend (up- and downregulated codDEGs).

### 3.6. Manipulated Levels of DJ-1 in Cells Indicate a Switch in Transcription and Cell Homeostasis

To isolate the direct impact of DJ-1 levels independent of X-ray treatment, we compared gene expression changes in DJ-1 KD cells versus the non-specific siRNA-RLUC control. Approximately 200 DEGs reflected a coordinated antiviral innate immune response (see details in [29]). Functional enrichment of the remaining genes, those specific to DJ-1 KD and not involved in the antiviral response, revealed associations with RNA metabolism and mitochondrial functions, with downregulated genes linked to cell viability and mitophagy [29]. These findings suggest that cells depleted of DJ-1 may become more susceptible to cell-death pathways and inflammatory signaling.

**Figure 5:**
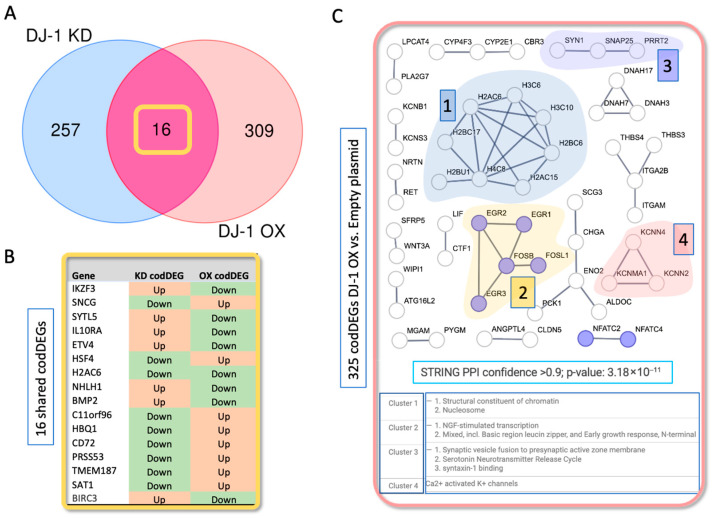
Analyzing the codDEG for DJ-1 OX, and DJ-1 KD. (**A**) Venn diagram showing the combined upregulated and downregulated codDEGs for DJ-1 OX versus empty plasmid and DJ-1 KD versus siRNA-RULC. (**B**) List of the 16 shared codDEGs from (**A**), highlighted by a yellow frame. The expression trend (up- or downregulation) in each cellular context is indicated. (**C**) STRING-based PPI networks (confidence score ≥ 0.9) generated from the 325 DJ-1 OX-specific codDEGs (pink circle in (**A**)). Only interaction networks containing at least two genes are shown. Colored nodes indicate the major functional enrichment category based on GO molecular function, including RNA polymerase II cis-regulatory region sequence-specific DNA binding (red nodes). Additional functional enrichment annotations are provided in Supplementary Figure S3.

Figure 5A shows the overlap between codDEGs in the DJ-1 KD and DJ-1 OX conditions in the absence of X-ray treatment. DEG analyses were performed by comparing each manipulated condition to its corresponding control (empty plasmid for DJ-1 OX and siRNA-RLUC for DJ-1 KD). The overlap between the OX codDEGs and KD codDEGs was limited to 16 shared genes (hypergeometric *p*-value 5 × 10^−4^). For 15 of these, the direction of regulation in KD versus OX was opposite, consistent with the expected contrasting cellular responses to reduced versus elevated DJ-1 levels (Figure 5B).

STRING-based PPI analysis of the >300 genes unique to DJ-1 OX (relative to empty plasmid) revealed small but distinct interaction networks (Figure 5C). The main clusters are color-coded, and their representative functions are listed (clusters 1–4; Figure 5C). GO molecular functional enrichment to regulation of RNA polymerase II, transcription factor activity, and DNA binding (Supplementary Figure S3) is modest (FDR 1.4 × 10^−3^ and moderate enrichment of 2.5 fold). Among the transcription factors suppressed in DJ-1 OX relative to empty plasmid were early-response genes such as EGR1 and FOSB, which mediate stress and inflammatory signaling into gene-expression changes. These enrichment results (Supplementary Figure S3) are consistent with transcriptional reprogramming triggered by excess DJ-1 in cells.

### 3.7. Induction of ncRNAs in Response to X-Ray Irradiation

The RNA-seq results show that about 19% of all transcripts are classified as ncRNAs (totaling 16,825 genes filtered by a minimal expression threshold; see Section 2). A notable observation is that ncRNAs generally exhibit very low expression levels (Supplementary Figure S4). Specifically, only 3.4% of the total expression is attributed to ncRNAs (3230 genes), while the remaining 96.6% corresponds to coding genes. Approximately 75% of ncRNAs show low expression (<2 TMM), 8% are expressed at ≥10 TMM. Partitioning these ncRNAs to their biotypes reveals that most belong to lncRNAs, many of which are novel or annotated as antisense (AS) transcripts. Other prominent biotypes include pseudogenes (PGs), both processed and unprocessed, reflecting origins from gene duplication or retrotransposition. Notably, a substantial fraction of PGs is transcribed, potentially exerting regulatory effects on their functional gene counterparts.

**Figure 6:**
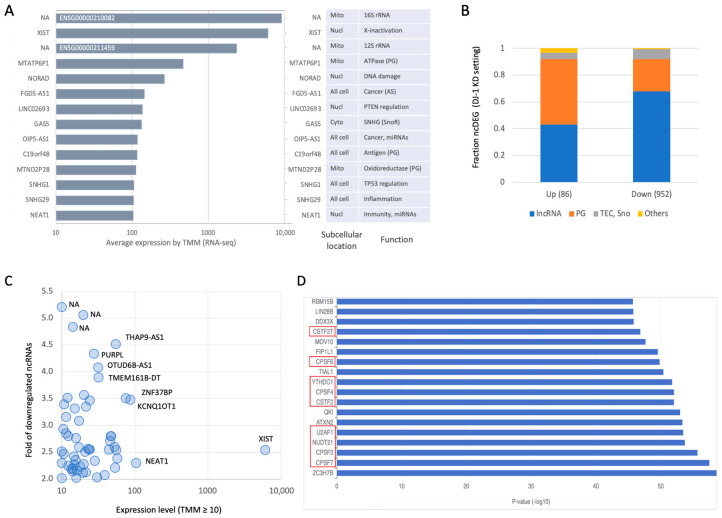
Quantitative summary of the ncRNAs by X-ray irradiation on DJ-1 KD cellular setting. (**A**) List of top expressing ncRNAs (≥100 TMM) along their subcellular localization and main function. (**B**) Partition of major biotypes of ncRNAs for the ncDEG of DJ-1 KD setting for up- and downregulated genes. Only genes with FC ≥|2| are included to secure robustness. (**C**) Scatter plot of the downregulated ncDEG (log scale, with TMM ≥10, on *x*-axis) and the inverse fold change in the expression level (4 means the expressing level is 0.25 of the matched control cells, *y*-axis). Several of the genes are labeled by gene symbol. (**D**) Statistically significant candidate coding genes according to the most significant 100 ncDEGs analyzed for the RNA-protein interaction category based on LncSEA 2.0. Red frames are set of proteins working together for pre-mRNA processing. List of the protein-interaction predicting results is accessible in Supplementary Table S4.

Figure 6A lists the 14 highly expressed ncRNAs (>100 TMM) including XIST, which regulates X-chromosome inactivation, and mitochondrial genes such as 16S and 12S rRNA. Among the highly expressed lncRNAs are GAS5 (also known as SNHG2), SNHG1, NORAD, and NEAT1, abundant transcripts implicated in cell-cycle control, cancer progression, DDR, and inflammation, respectively [34]. Examples of several ncDEGs from all settings and across different initial abundance are shown in RNA-seq and RT-PCR (Supplemental Figure S5). The composition of the 1038 ncDEGs associated with DJ-1 KD (using a strict FC threshold of ≥|2|) is shown in Figure 6B. The fraction of PGs among the upregulated ncDEGs in the DJ-1 KD condition is 2.5-fold higher (Figure 6B). Among the downregulated PGs, approximately 30% are related to ribosomal and mitochondrial proteins, while the majority (70%) remain uncharacterized.

We therefore focused on downregulated ncDEGs with modest baseline expression (≥10 TMM) and substantial downregulation (≥2-fold). Figure 6C shows that among this set (56 ncRNAs), several are stable ncRNAs (e.g., XIST, NEAT1). Additional ncRNAs in this group have been studied in the context of cancer (e.g., THAP9-AS1, PURPL, OTUD6B-AS1), transcriptional regulation (e.g., KCNQ1OT1), and chromatin structure.

### 3.8. X-Ray Irradiation on siRNA DJ-1 KD Cells Resulted in Dysregulation of Coding and ncRNA Transcription

External X-ray irradiation led to increased DSBs and elevated γ-H2AX levels (Figure 1A,D). We next tested whether DJ-1 depletion by siRNA affected the expression of genes involved in the DNA damage response (DDR). To this end, we compiled a set of key DDR genes, including ATM, PRKDC, XRCC5, XRCC6 (KU70/80), and RAD51, along with their immediate interaction partners, generating a collection of 46 genes (Supplementary Table S5). Analysis of the top codDEGs in DJ-1 KD cells revealed nine overlapping genes (BRCA2, ATRX, LIG4, NBN, MRE11, XRCC4, ATM, DCLRE1C, and TP53). Statistical testing using a 2×2 Fisher exact test indicated a 3.7-fold enrichment of DDR genes, with a hypergeometric *p*-value of 1.8 × 10^−3^ for ≥9 overlapping genes. Notably, BRCA2, ATRX, and LIG4, which participate in DDR and chromatin maintenance, were strongly downregulated (6.7-, 5.5-, and 3.8-fold, respectively). Interestingly, TP53 was the only upregulated gene among these nine (2.5-fold), potentially acting as a primary DNA damage sensor. The suppressed expression of core DDR genes supports the notion that reduced levels of DDR components may impair downstream DNA repair signaling. Further experiments are required to clarify a direct role of DJ-1 in coordinating DDR machinery.

Given the complex and cell state-specific regulatory network between ncRNAs and coding genes, we employed multiple computational tools (see Section 2) to generate a ranked list of proteins likely affected by ncDEGs (Figure 6D). Notably, candidate proteins influenced by lncRNA changes included a set of RNA-binding proteins that coordinately regulate 3’ RNA processing and pre-mRNA splicing (Figure 6D). Specifically, NUDT21 is a component of 3’ RNA cleavage and polyadenylation processing. A group of CSTF genes (red frames, Figure 6D) act as activators of pre-mRNA 3’-end cleavage and polyadenylation, processes essential for mRNA maturation. Many of these nuclear RNA-binding proteins regulate 3’ end cleavage of mRNAs and, consequently, indirectly influence miRNA biogenesis (e.g., ZC3H7B). For a detailed summary of the enrichment analysis, see Supplemental Table S4.

### 3.9. Potential Functions of ncRNAs as Antisense in Response to X-Ray Irradiation

**Figure 7:**
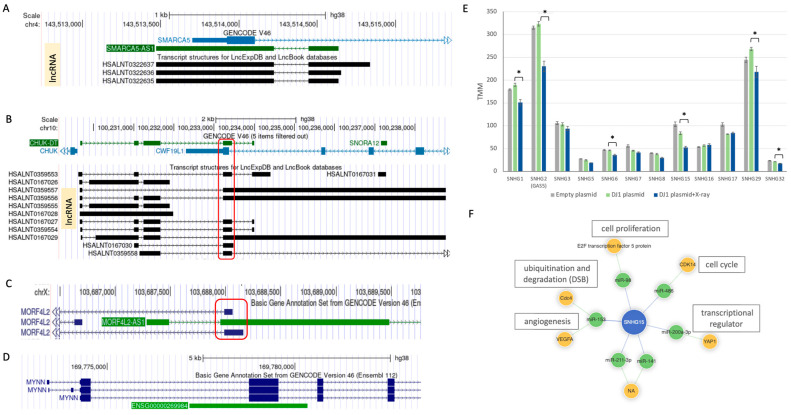
Functional candidates of ncDEG in the DJ-1 OX following X-ray. Genome browser view according to GENCODE V46. Examples of lncRNAs as antisense (AS) and the overlap on exon boundaries are shown. (**A**) Transcription overlapping of SMARCA5-AS1 and SMARCA5. The transcripts overlap the 3’-exon by complementation. (**B**) Transcription overlapping of CHUK-DT. In red frame, the overlap in exon boundaries with CWF19L1 in the complementary strand. (**C**) Transcription overlapping of MORF42L2-AS1 and MORF42L2. In red frame, the overlap of the first exon with the complementary strand. (**D**) Transcription overlapping of ENSG00000269984 and several versions of MYNN. (**E**) Relative expression of 14 members of SHNGs with ≥20 TMM. The majority of the SHNGs were downregulated 6 h after X-ray exposure. There are 6 of the genes in which the *p* values (*t*-test) were significant (marked by asterisk). The results from empty vector (gray) show no statistical significance relative to DJ-1 OX cells. Details are available in Supplementary Table S6. (**F**) The interactome of SNHG15 (based on lncSEA 2.0) lists the miRNA direct interactors and their key protein targets that potentially impact the listed cellular processes.

Figure 7 presents several candidate ncDEGs identified in the DJ-1 OX setting with the following functions: (i) antisense (AS) RNAs that impact transcriptional regulation; (ii) members of the SNHG family, which serve as host genes for snoRNAs; and (iii) lncRNA-protein candidates. Figure 7A highlights how the lncRNA SMARCA5-AS1 might regulate SMARCA5 transcription by competing with the 5’ exon of the transcript. SMARCA5 (SNF2H) is an essential ATPase involved in chromatin remodeling and plays a key role in the DDR [35]. SMARCA5 depletion increases sensitivity to ionizing radiation and impairs DSB repair [36]. It is possible that it facilitates access to damaged DNA for major repair mechanisms.

CHUK-DT is an upregulated lncRNA that complements the CWF19 family member (Figure 7B). CHUK-DT consists of a diverse collection of alternatively spliced ncRNAs, several transcribed in the opposite direction, potentially interfering with transcription. CWF19L1 (CWF19-like cell cycle control factor 1) overlaps with the exon sequence (Figure 7B). Its overexpression has been shown to inhibit the G1/S phase transition by suppressing cell cycle proteins such as CDK4 and CDK6 [37].

Figure 7C shows MORF4L2-AS1, which may act as an antisense regulator of MORF4L2. Its downregulation could result in increased levels of MORF4L2, a gene involved in positive regulation of transcription by RNA PolII, heterochromatin assembly, and histone modification. Figure 7D illustrates potential transcriptional interference for MYNN, a gene involved in immune responses and inflammation, which contributes to the control of gene expression.

### 3.10. Suppression of SNHG Family Members Following X-Ray Irradiation in DJ-1 OX Cells

Among the highest-expressing ncRNAs whose functions have been previously studied (≥100 TMM, Figure 6A), several belong to the SNHG family (note that GAS5 is also called SNHG2). We confirmed the statistical stability of these examples relative to controls (Supplemental Figure S6). GAS5 was downregulated by X-ray in DJ-1 OX cells. However, its expression level was unchanged between DJ-1 OX cells and cells transfected with an empty plasmid (Supplemental Figure S6). Functionally, GAS5 localizes to mitochondria, where it helps maintain energy homeostasis [38]. All listed examples (Supplemental Figure S6) showed a similar trend. Many of the identified ncDEGs are also regulated by miRNAs. For instance, GAS5 is negatively regulated by miR-21 [39], while MINCR, which facilitates expression of MYC-target genes crucial for cell cycle progression, is modulated by miR-146b-5p in the context of NF-κB signaling and inflammation [40].

We focused on lncRNAs of the SNHG family (Small Nucleolar RNA Host Genes, Figure 7E). These genes serve as hosts for snoRNAs (small nucleolar RNAs), which play key roles in rRNA modification and ribosome biogenesis. We identified a total of 21 SNHG genes, accounting for ~8% of total ncRNA expression in cells. Among significantly downregulated genes, SNHGs represent ~24% of all ncRNA genes in the DJ-1 OX setting (Supplementary Table S2).

Figure 7E shows the expression of 12 SNHG family members (with a minimal expression level of ≥20 TMM), among which six were significantly suppressed. For example, SNHG15 expression was reduced to 62% of its baseline. GAS5 (SNHG2), SNHG29, and SNHG1 were downregulated to 71%, 81%, and 80% of their pre-irradiation levels, respectively, in DJ-1 OX cells exposed to X-ray. Across different cell types, SNHGs have been shown to modulate gene expression by sponging miRNAs [41,42], regulating gene expression, cell cycle, and cancer progression [34]. Figure 7F illustrates the interactome of SNHG15 known to regulate inflammatory markers (e.g., p65, TNF-α) [43]. Due to its high cytoplasmic abundance, SNHG15 may shift miRNA availability. Direct interactions with miRNAs (e.g., miR-486, miR-200-3p) influence multiple cellular responses, including proliferation and cell cycle regulation. This interactome aligns with observations that suppression of SNHG15 leads to cell cycle arrest in cancer models. We propose that the coordinated suppression of SNHGs following X-ray exposure results from both direct and indirect effects of DJ-1.

### 3.11. Cells with Overexpressed or Depleted DJ-1 Resulted in an Inverse Expression Trend

We investigated whether any ncDEGs act as molecular hubs responding to X-ray in the contexts of DJ-1 OX and DJ-1 KD. A Venn diagram of ncDEGs for N.T., DJ-1 KD, and DJ-1 OX revealed minimal overlap among ncRNAs (Supplementary Figure S7). The set of ncDEGs defining the X-ray response in DJ-1 KD was almost entirely unique (98.6%), and no ncRNAs were shared across all cellular conditions. This pattern is consistent with observations for codDEGs, in which genes were largely specific to each cellular setting. Nevertheless, 12 ncRNAs were shared between the DJ-1 OX and DJ-1 KD conditions (Supplementary Figure S7).

Table 1 shows that most ncDEGs common to both DJ-1 OX and DJ-1 KD settings are classified as antisense lncRNAs (L-AS) or pseudogenes (PG). To investigate whether processed PGs might function as transcriptional regulators via hybridization, we conducted a BLAST search against the entire transcriptome, identifying genes with high-confidence matches to coding transcripts (BLAST E-score < 10^−20^). We found that most PGs can successfully hybridize either to similar PGs (if present) or to their gene of origin (Table 1). While experimental validation is needed, we propose that the roles of L-AS ncDEGs may influence mRNA dynamics (e.g., INTS6-AS1), chromatin structure (e.g., ATXN1-AS1), transcription regulation (e.g., MYNN antisense), and the chromatin–transcription axis (e.g., SMARCA5-AS1). Table 1 also shows that the trends of upregulation (U) and downregulation (D) were reversed between OX and KD settings for 8 of the 12 genes. All five genes listed in Table 1 that were upregulated in the DJ-1 OX setting exhibited an inverse expression trend in the DJ-1 KD setting. These genes with opposing expression patterns may represent direct links to DJ-1 cellular status.

### 3.12. Enrichment of Cell Cycle Arrest and Chromatin Functional Classes Under Varying DJ-1 Expression Levels

Most of the reported lncRNAs (Supplementary Table S2) are low-expressing and poorly annotated, making it difficult to assign specific functions to individual transcripts. Therefore, we evaluated their potential roles at the level of the ncRNA set rather than focusing on single genes. To this end, we applied an enrichment analysis (using LncSEA 2.0) to the collection of ncRNAs that were downregulated by X-ray in the DJ-1 OX setting. Of the 81 most significant ncRNAs, 55 could be uniquely mapped for downstream analysis (Supplemental Table S4, see Section 2).

Several classes of chromatin regulators and histone modifications were enriched among the downregulated genes in the DJ-1 OX cellular setting (Table 2). FAM66C, FLJ37453, and SNHG10 were identified by ChIP-seq as chromatin-associated regulators (adjusted *p*-value = 3.5 × 10^−4^). This finding is consistent with the observation that 50% of all downregulated, uniquely mapped lncRNAs (25 genes) were known to bind the H3K4me2/3 histone marks (adjusted *p*-value = 9 × 10^−23^; Table 2). Another enriched histone modification was H3T11P, a marker corresponding to phosphorylation at Thr11 of histone H3 (adjusted *p*-value = 5 × 10^−16^). These results suggest that many of the downregulated lncRNAs may influence gene-expression programs through interactions with specific histone epigenetic marks.

Table 2 also highlights numerous experimentally validated functional sets (from LncTarD 2.0), including categories related to cell growth, cancer progression, and cell cycle regulation (adjusted *p*-values < 2 × 10^−5^). The eight ncDEGs associated with cell proliferation include BCYRN1, FOXCUT, HOXA11-AS, LINC01535, MINCR, MNX1-AS1, SNHG10, and SNHG15. When evaluating the downregulated lncRNAs as a group, several recurrent target genes emerged within the category of RNA–protein interactions. Examples include DDX3X, ATXN2, and CSTF2T, with 26 of the 55 mapped lncRNAs linked to these proteins (adjusted *p*-values ranging from 6.6 × 10^−32^ to 2.8 × 10^−28^). These proteins are multifunctional regulators involved in RNA metabolism, cell cycle control, innate immunity, apoptosis, and translation.

Notably, DDX3X also directly participates in the DNA damage response and contributes to genome stability, whereas ATXN2 and CSTF2T primarily influence cytoplasmic mRNA processing. ATXN2 associates with polysomes and interacts with the poly(A)-binding protein PABPC1 to regulate translation initiation and mRNA decay. CSTF2T functions in pre-mRNA cleavage required for polyadenylation.

Together, the analysis suggests that following X-ray exposure, genome stability is compromised, and that the alterations in lncRNA expression may modulate the activity of RNA metabolism, enabling cells to adapt to the altered cellular state.

## 4. Discussion

In this study, we assessed how cells respond to X-ray irradiation under varying levels of DJ-1 by examining the differential transcriptomic landscape. Most studies on DJ-1 focus on its role in sensing and responding to oxidative stress [44]. The cellular protective function of DJ-1 is attributed in part to its activity in the mitochondria, where it elevates the expression of uncoupling proteins, suppresses ROS production, and inhibits apoptosis [45]. At the transcriptional level, DJ-1 stabilizes Nrf2, enhancing its activity as a transcription factor for antioxidant genes [5].

The notion that DJ-1 might activate alternative mechanisms predisposing cells to pathological states has also been proposed [1,46]. We show that DJ-1 influences the transcriptomic landscape following genotoxic stress, highlighting its nuclear role. A recent study identified DJ-1 as a regulator of PARP1, linking oxidative-stress sensing to DNA repair in Parkinson’s disease [23]. X-ray treatment induces DSBs, monitored here through the rapid kinetics of γ-H2AX accumulation, an essential step in both HR and NHEJ repair pathways [47]. The irradiated cells recovered successfully with minimal cell death.

To isolate the specific impact of DJ-1, we analyzed functional enrichments associated with altered DJ-1 expression. Cells treated with non-specific siRNA (siRNA RULC) served as controls for the DJ-1 KD setting, while empty vector-transfected cells served as controls for DJ-1 OX. DJ-1 overexpression and depletion were validated at the protein level in the OX (Figure 1C) and KD (Figure 2B) settings, respectively. While HEK293 cells allow robust genetic manipulation, they may only partially recapitulate neuronal or cancer contexts, underscoring the need for follow-up studies in more relevant models. Notably, in contrast to the robust antiviral response triggered by siRNA manipulation, no antiviral-like response was observed 6 h after X-ray exposure [29]. We did not examine the kinetics of DNA repair, which vary by tissue and cell type [48].

A key finding in the DJ-1 OX setting is the downregulation of ribosomal genes following X-ray irradiation, including mitochondrial ribosomal genes and oxidative phosphorylation pathways (Figure 4F). Ribosome assembly requires coordinated RNA polymerase activities, rRNA processing, RNA modifications, and ribonucleoprotein assembly. Because ribosome biogenesis consumes substantial cellular resources, its regulation is essential for homeostasis. The observation that dozens of ribosomal transcripts are downregulated by X-ray in DJ-1 OX cells aligns with the vulnerability of unassembled ribosomal components, which can trigger ribosomal stress [49]. The intimate link between ribosome biogenesis and cell growth underscores the central role of p53 signaling in stress responses [50,51]. The interaction between DJ-1 and p53 has been documented in the contexts of PD and cancer [5]. After X-ray exposure and DSB induction, p53 activates cell-cycle arrest and DNA repair, with its activity tightly regulated by MDM2 [52]. Although requiring further validation, the disruption of ribosome biogenesis (i.e., nucleolar stress) observed in DJ-1 OX cells may activate the MDM2–p53 stress response. Ribosomal proteins such as RPL5, RPL11, and RPL23 can directly bind MDM2, stabilizing p53 [53]. Thus, elevated DJ-1 under genotoxic stress drives a transcriptional state characterized by reduced ribosome biogenesis and translation, reflecting a stress-adaptation strategy that conserves resources and supports genome stability. Reduced mitochondrial function may further contribute to this state. Active mitochondria are a major source of ROS. Because X-ray irradiation increases ROS, the downregulation of oxidative phosphorylation genes in DJ-1-high cells may represent a protective mechanism to limit additional oxidative burden. DJ-1 KD triggered inflammatory and oxidative stress-related responses, including significant NEAT1 upregulation (2.2-fold, q = 3.7 × 10^−5^), which eventually leads to sustained STAT1 phosphorylation [29]. Overall, DJ-1 appears to promote transcriptional programs that support homeostasis under genotoxic stress.

We also assessed the impact of X-ray exposure in the DJ-1 OX and DJ-1 KD settings by examining ncDEGs, particularly lncRNAs. ncDEGs contributed significantly to the observed transcriptomic alterations. Approximately 25% of all detected ncRNAs met ncDEG criteria (log_2_FC > |1|), a proportion likely reflecting reduced cellular resilience under near-complete DJ-1 depletion [29]. Several observations support this conclusion: more than 1000 lncRNAs changed expression within 6 h of irradiation, 92% of which were downregulated; and ncDEGs in DJ-1 KD and DJ-1 OX cells were nearly disjoint (Figure 5B).

One striking pattern is the highly coordinated suppression of SNHG transcripts in DJ-1 OX cells following X-ray exposure. SNHG genes are associated with cancer progression [54]. Among the 12 of 21 SNHG family members with high expression (≥20 TMM), half were significantly downregulated (Figure 7E). SnoRNAs, which mediate rRNA modifications, are hosted within SNHG introns and also reside within ribosomal protein genes [55]. Thus, the downregulation of both SNHG genes and ribosomal proteins suggests ribosome destabilization via direct effects on rRNA processing. Additionally, SNHG genes such as SNHG15 affect miRNA sponging and thereby post-transcriptional regulation. SNHG15, a host of SNORA9, functions not only in snoRNA production but also as a miRNA hub (Figure 7F). Although speculative, DJ-1 activity may indirectly affect miRNA profiling by altering damage-induced RNAs generated at DSB sites [56], which influence miRNA biogenesis. miRNA-mediated regulation shapes survival pathways beyond canonical DNA repair [57]. These findings point to additional layers of regulation that warrant future investigation.

Several lncRNAs upregulated in DJ-1 OX cells after X-ray exposure function in transcriptional or translational regulation. PAIP1P1, derived from PAIP1, may influence translational initiation; ENSG00000229447, a pseudogene related to GTF2A2, may modulate transcription initiation fidelity. Other ncDEGs participate in homeostatic processes such as glycolysis (TPI1P2), protein folding (CCT4P2), and mRNA stability (BCLAF1P2). Although direct experimental validation is lacking, we propose that lncRNAs contribute to the complexity of stress responses by shifting homeostasis and modulating pathways such as p53 activation [58,59]. SMARCA5-AS1, involved in chromatin remodeling and genome integrity [60], dynamically responds to DJ-1 levels and X-ray exposure (Table 1), indicating potential roles in chromatin architecture and transcriptional regulation.

DJ-1 OX cells display restricted but robust transcriptional changes after X-ray exposure. Elevated DJ-1 levels in cancer are associated with advanced stages and poor prognosis [61]. Yet the mechanisms by which DJ-1 coordinates oncogenic signaling remain incompletely understood [46]. For example, DJ-1 promotes cyclin-D1 expression in colorectal cancer, facilitating cell-cycle progression, and suppresses p53-mediated apoptosis under genotoxic stress [62]. In contrast, DJ-1 KD cells show widespread transcriptomic alterations driven by lncRNAs, affecting the cell cycle, proliferation, mRNA maturation, miRNA stability, and RNA-dependent nuclear functions (Table 2).

## 5. Conclusions

This study demonstrates that DJ-1/PARK7 is a central mediator linking redox regulation to the transcriptional response following X-ray-induced DNA damage. In naïve cells, X-ray exposure elicited only a modest DNA damage response, whereas altering DJ-1 expression profoundly reshaped gene expression programs. DJ-1 overexpression induced broad repression of ribosomal, mitochondrial, and chromatin-associated genes, accompanied by marked downregulation of lncRNAs and small nucleolar RNA host genes (SNHGs). Conversely, DJ-1 knockdown resulted in extensive and nonspecific transcriptional dysregulation, reflecting a breakdown in global transcriptional control and cellular homeostasis.

These opposing outcomes underscore DJ-1’s essential role in balancing redox signaling, RNA metabolism, and genome stability. By coordinating both coding and non-coding RNA responses under genotoxic stress, DJ-1 emerges as a key integrator of antioxidant defense and transcriptional homeostasis. Its dual influence on redox pathways and transcriptional networks positions DJ-1 as a potential therapeutic target in conditions marked by oxidative stress and impaired DNA repair.

## Figures and Tables

**Table 1:** ncDEG shared between DJ-1 OX and DJ-1 KD cellular settings.

Ensembl	Gene Name	Type ^a^	Gene Description	# Exons	Size (nt) ^c^	Hyb ^b^	Trend OX	Trend KD
ENSG00000225569	CCT4P2	PG	chaperonin w TCP1 subunit 4 PG2	3	1548	S	**U**	**D**
ENSG00000234084	NA	Lnc	Novel	3	577	C	**U**	**D**
ENSG00000242600	NA	TPG	mannose binding lectin 1, PG	4	758	-	**U**	**D**
ENSG00000245112	SMARCA5-AS1	L-AS	SMARCA5 AS RNA 1	2	1149	C	**U**	**D**
ENSG00000269984	NA	L-AS	novel, AS of MYNN	2	3143	C	**U**	**D**
ENSG00000198221	NA	L-DT *	AFDN DT	2	4095	S	D	D
ENSG00000218426	RPL27AP6	PG	60S ribosomal protein L27a PG	1	447	S	**D**	**U**
ENSG00000226415	NA	PG	triosephosphate isomerase 1 PG1	1	750	S	**D**	**U**
ENSG00000229931	NA	L-AS	ATXN1 AS RNA 1	3	1913	-	D	D
ENSG00000231154	NA	L-AS	MORF4L2 AS RNA 1	3	2086	C	D	D
ENSG00000236778	INTS6-AS1	L-AS *	INTS6 AS RNA 1	2	4305	-	D	D
ENSG00000276168	RN7SL1	mis.	RNA of signal recognition particle	1	299	-	**D**	**U**

^a^ TPG, transcribed pseudogene; Lnc, L-, long non-coding RNA; AS, antisense; mis., miscellaneous; *, multiple transcripts. ^b^ Hyb, hybridization by BLAST E-value < 1 × 10^−20^. Match to same strand direction (S), and the complementary strand (C). ^c^ Size, in case of multiple transcripts, the longest/representative one is reported.

**Table 2:** Functional enrichments of downregulated genes from DJ-1 OX cellular setting by LncSEA2.0.

Class (LncSEA)	Set	# (Query)	Size of Set	*p*-Value	Adj. *p*-Value	Jaccard	Simpson ^a^
RNA Histone Modification	H3K4me2-3	25	1802	1.80 × 10^−23^	9.00 × 10^−23^	0.014	0.455
	H3T11P	20	1863	1.84 × 10^−16^	4.60 × 10^−16^	0.011	0.364
	H3K122ac	20	1991	6.48 × 10^−16^	1.08 × 10^−15^	0.010	0.364
	H2AFX	6	945	2.59 × 10^−4^	3.24 × 10^−4^	0.006	0.109
Chromatin Regulators	NCOA1	3	48	1.32 × 10^−5^	3.99 × 10^−3^	0.030	0.063
Experimental Validated Function	cancer progression	7	307	1.68 × 10^−8^	9.07 × 10^−7^	0.020	0.127
	cell metastasis	6	264	1.93 × 10^−7^	6.94 × 10^−6^	0.019	0.109
	cell cycle	5	143	2.57 × 10^−7^	6.94 × 10^−6^	0.026	0.091
	proliferation	8	838	1.16 × 10^−6^	2.51 × 10^−5^	0.009	0.145
RNA Protein Interaction	DDX3X	26	961	6.65 × 10^−32^	6.69 × 10^−28^	0.026	0.473
	ATXN2	26	1032	4.20 × 10^−31^	2.11 × 10^−27^	0.025	0.473
	CSTF2T	26	1328	2.75 × 10^−28^	9.23 × 10^−25^	0.019	0.473

^a^ Simpson assesses the diversity in the functional roles of lncRNAs in the database.

## Data Availability

The raw data was submitted to ArrayExpress Accession E-MTAB-14761. Detailed summary of the quality control and reads assignment is provided in Supplementary Table S1.

## Supplementary Materials

The following supporting information can be downloaded at: https://www.mdpi.com/article/10.3390/cells14231860/s1. All processed and normalized data produced in this study are available in Supplementary Tables and Figures. Table S1: Quality control of NGS results. Table S2: DEG of X-ray in the N.T., OX and KD settings. Table S3: Enrichment significant DJ-1 KD. Table S4: Protein-RNA interactions from LncSEA 2.0. Table S5: List of 46 genes of the DDR unified set. Table S6: List of SNHG members, Figure S1: X-ray radiation and cell survival. Figure S2: Network analysis and functional enrichment of the codDEGs from the DJ-1 KD setting. Figure S3: Enrichment of codDEGs based on STRING-PPI analysis of DJ-1 OX. Figure S4: Statistics of ncRNAs from the N.T. cells. Figure S5: Expression of lncRNAs across all cellular settings. Figure S6: Analyzing the family members of SNHG family ncRNAs. Figure S7: Venn diagram of the ncDEG for N.T., DJ-1 OX, and DJ-1 KD.

## Abbreviations

DDR	DNA damage response
DEG	differentially expressed genes
DSB	double-strand break
HR	homologous recombination
KD	knockdown
NGS	Next-generation sequencing
N.T.	not treated
ncRNA	non-coding RNA
NHEJ	non-homologous end joining
Nt	nucleotides
OX	overexpression
PCA	principal component analysis
PD	Parkinson’s disease
PPI	protein–protein interaction
ROS	reactive oxygen species
RT	room temperature
TF	transcription factor
TMM	trimmed mean of the M-values normalization
