# Supplementary material for: Manipulating PARK7/DJ-1 Levels by Genotoxic Stress Alters Noncoding RNAs and Cellular Homeostasis
Source: Cells. 2025 Nov 25;14(23):1860. doi: 10.3390/cells14231860 (PMC12691451; doi:10.3390/cells14231860)
Supplement: Supplementary file 1 [file cells-14-01860-s001.zip › cells-3980363-figures.pdf]

Manipulating PARK7 / DJ-1 Expression Levels Under Genotoxic Stress Alters Noncoding RNAs and Modulates Cellular Homeostasis

Keren Zohar[1], Haya Zoubi[2], Michal Goldberg[2], Tsiona Eliyahu[1], Michal Linial[1,*]

Supplementary Figures

Figure S1

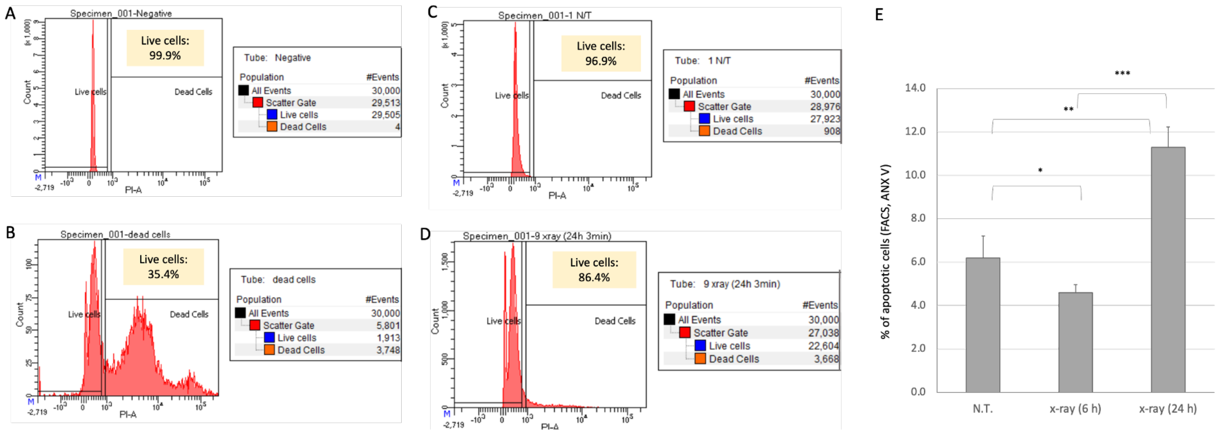

Figure S1. *X-ray radiation and cell survival. Representative results from N.T. cells and measuring of the PI staining (gating thresholds are marked by the vertical lines).* ***(A)*** *Untreated cells were used to assess cell viability.* ***(B)*** *A control that derived cell death (65.5% death cells) confirmed the sensitivity of the FACS method.* ***(C)*** *A representative result N.T. cells* ***(C)*** *cells.* ***(D)*** *Cells treated by X-ray of 10 GY and monitoring the irradiated cells 24 hours after the irradiation protocol. The fraction of live cells and the actual number of analyzed cells are shown. Each experiment of HEK293 cells included 30,000 cells. Partition to live and dead cells is based on FACS analysis using propidium iodide (PI) for staining.* ***(E)*** *Average of triplicates for each condition stained with Annexin V fluorescence as marker for apoptotic cells. There was a small difference between the N.T. and X-ray 10 GY at 6 hours. However, 24 h after irradiation the levels of cells' apoptosis was significantly increased. Significant difference is marked with *, p-value <0.05; **, p-value <0.01. *** p-value <0.005).*

Figure S2

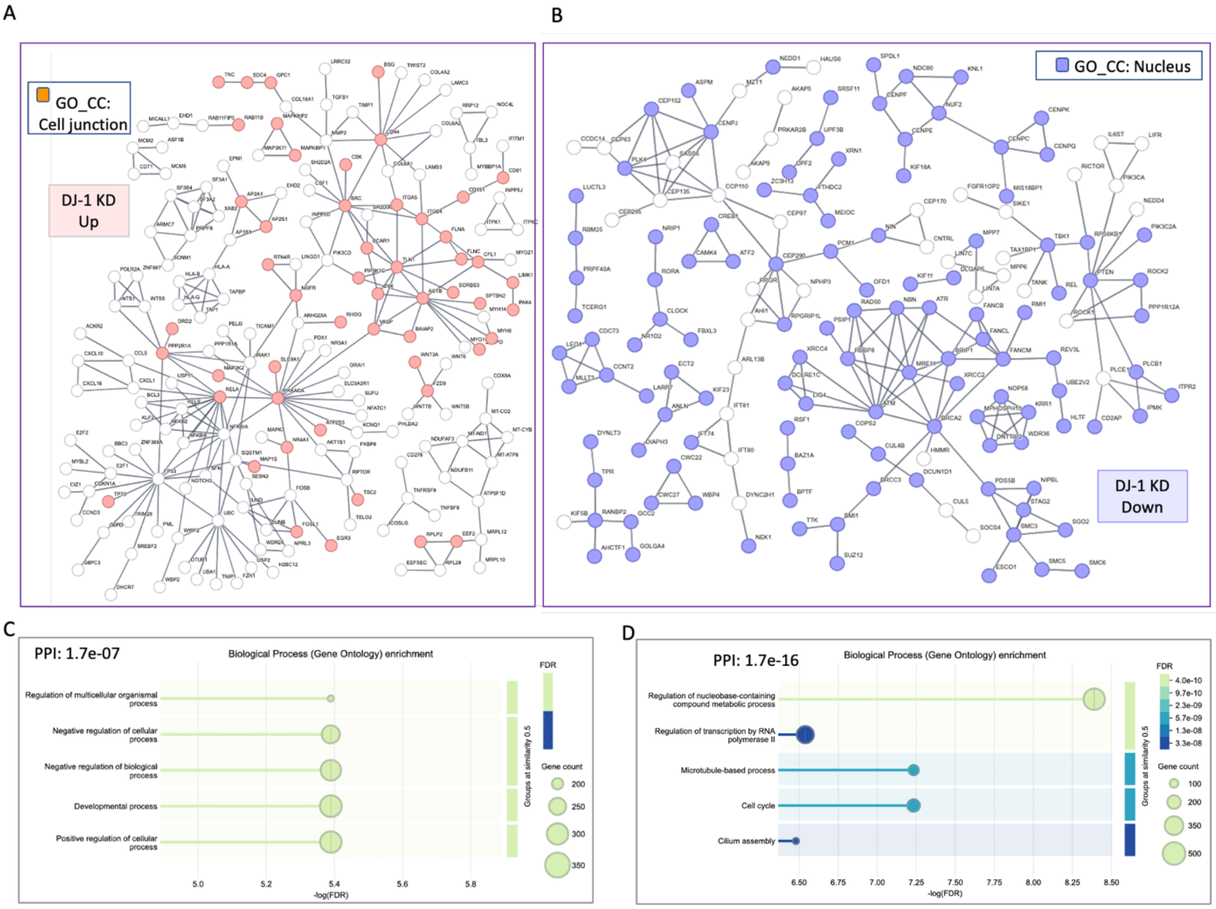

Figure S2. *Network analysis and functional enrichment of codDEGs from the DJ-1 KD setting.* ***(A)*** *STRING-based PPI networks (confidence score ≥0.9) for the DEGs by the purple-colored frames shown in* ***Figures 4A-3B.*** *Only networks of ≥3 genes are shown. The networks based on 775 genes for the upregulated (Up, left) and* ***(B)*** *the most significant 775 genes downregulated genes (Down, right). The colored nodes mark the most significant functional enrichment by GO cellular component (GO_CC) for Cellular junction (Up, left) and Nucleus (Down, right).* ***(C)*** *Main functional enrichment of GO_BP (Biological process using STRING visualization). The PPI network confidence is 1.7e-07.* ***(D)*** *Main functional enrichment of GO_BP (Biological process using STRING visualization) for the downregulated genes. Note that the difference in x axis for the -log10(FDR) enrichment scale.*

Figure S3

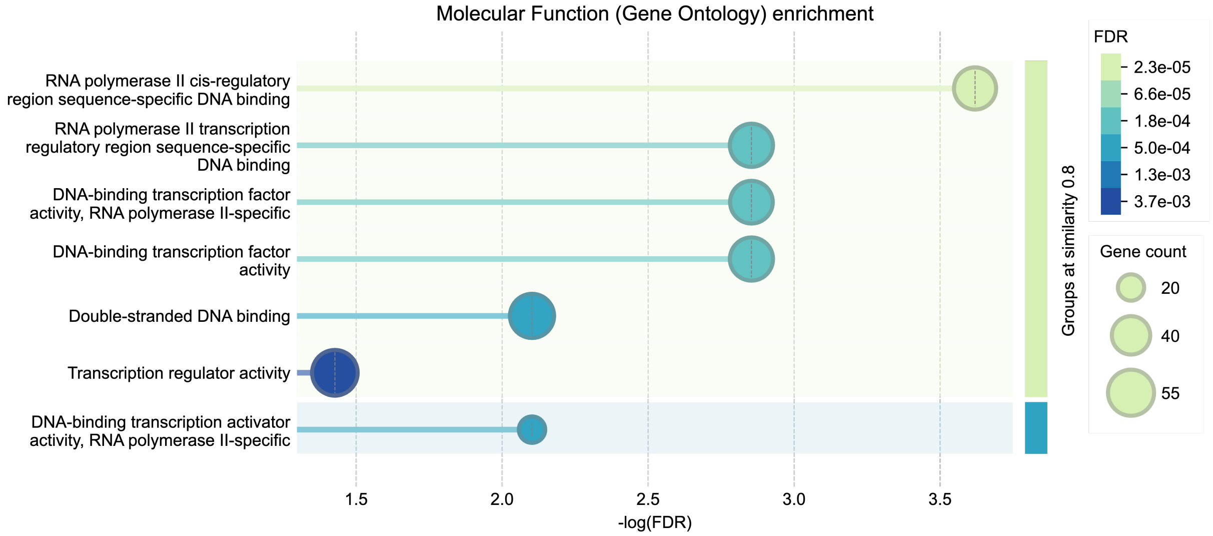

Figure S3. *Enrichment results of 325 codDEGs based on STRING-based PPI analysis of DJ-1 OX (relative to empty plasmid). GO molecular function enrichment identified annotations related to regulation of RNA polymerase II, transcription factor activity, and double strand DNA binding.*

Figure S4

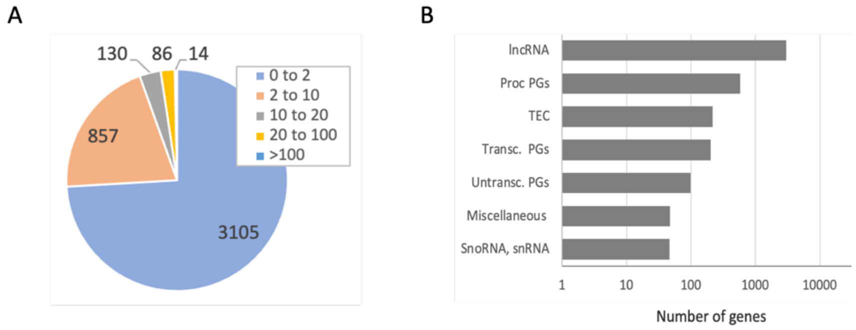

Figure S4. *Statistical view of ncRNAs from the N.T. cells.* ***(A)*** *The level of expression to set of genes according TMM normalization.* ***(B)*** *Partition of the amounts of genes according to the ncRNA biotypes (in log scale).*

Figure S5.

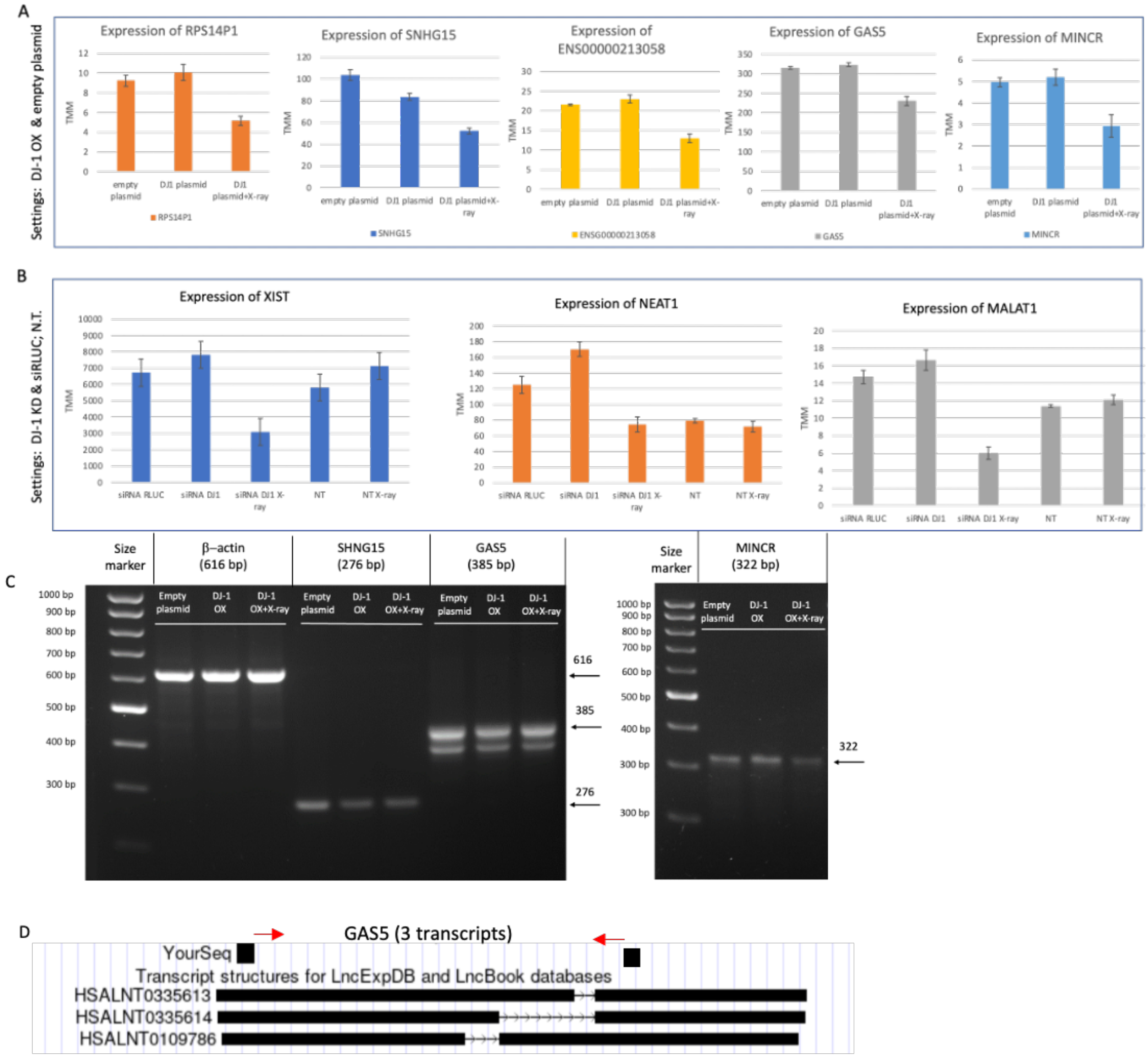

Figure S5. *Expression of lncRNAs across all cellular settings. The level of expression to is according to the TMM normalization. (A) A view of ncRNAs from the DJ-1 OX with and without X-ray irradiation. A control of empty plasmid is included. (B) A view of ncRNAs from the DJ-1 KD with and without X-ray irradiation. A control of siRNA of RULC (i.e., cells with non-specific siRNA) is included. The setting of N.T is shown with and without X-ray irradiation. In all the examples, the p value of the N.T. following X-ray was insignificant. Note the large difference in the y-axis between the high expressing lncRNAs (e.g., XIST) and the low expressing MINCR.*

Figure S6

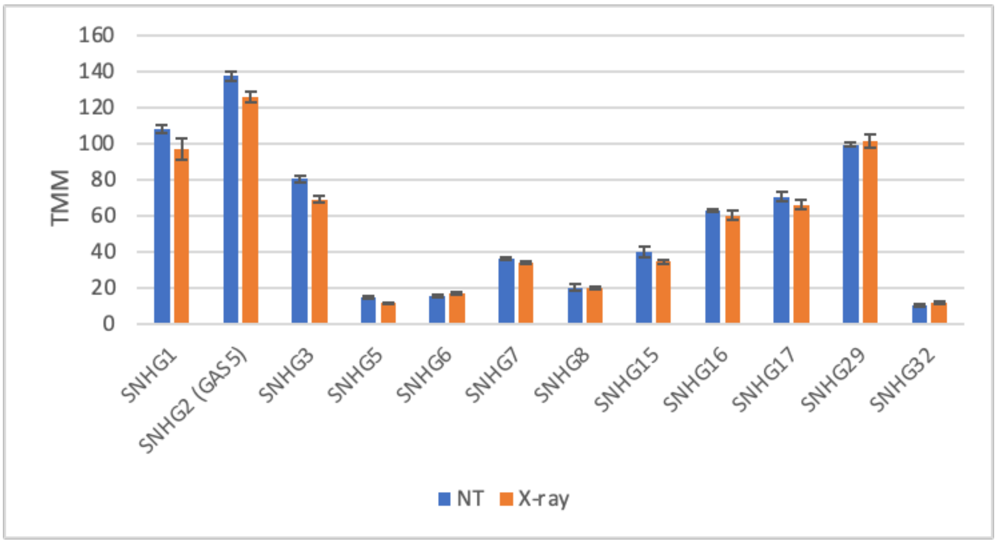

Figure S6. *Analyzing the family members of SNHG family ncRNAs. The measurements are based on N.T., and N.T + X-ray treated cells. Results and standard deviation are according to RNA-seq (in triplicates).*

Figure S7

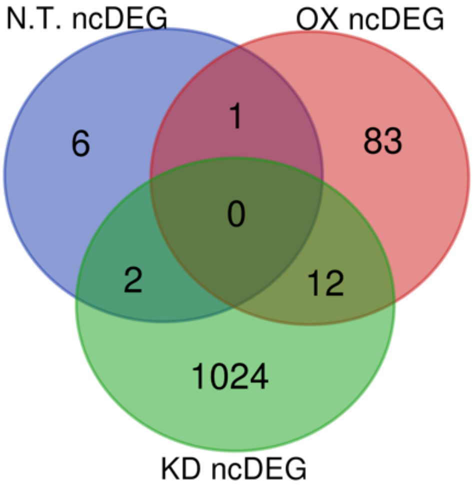

Figure S7. *Venn diagram of the ncDEG for N.T., DJ-1 OX, and DJ-1 KD.* ***(A)*** *Venn diagram of the ncDEG. The number of shared genes between any of the three cellular settings are indicated.*
